# Fat Mass Is Associated with Aging Rather than Menopausal Transition

**DOI:** 10.3390/healthcare14030333

**Published:** 2026-01-28

**Authors:** Carmen Gabriela Barbu, Irina Manuela Nistor, Alice Albu, Sorina Carmen Martin, Theodor Eugen Oprea, Anca Elena Sirbu, Adelina Vlad, Simona Fica

**Affiliations:** 1 Department of Endocrinology, Carol Davila University of Medicine and Pharmacy, 020021 Bucharest, Romania; 2 Department of Endocrinology, Elias Emergency University Hospital, 011461 Bucharest, Romania; 3 Department of Endocrinology and Metabolism, The National Institute of Endocrinology “CI Parhon”, 011863 Bucharest, Romania; 4 Division of Physiology and Neuroscience, Department of Functional Sciences I, Carol Davila University of Medicine and Pharmacy, 050474 Bucharest, Romania

**Keywords:** body composition, body weight, menopause, aging

## Abstract

**Background/Objectives**: Midlife is associated with changes in body weight and fat distribution in women; however, it remains unclear whether these changes can be attributed to chronological aging, menopause, or associated lifestyle changes. The objective of this study was to compare the possible differences in body fat distribution parameters measured by regional Lunar osteodensitometry scans (DXA) between clinically healthy, BMI-matched pre- and postmenopausal women. **Methods:** A cross-sectional analysis of body composition parameters, such as total body, android, and gynoid fat percentage, was performed using DXA hip and lumbar scans in 171 women aged 45–55 years. Comparisons were made across 50 premenopausal (median age 47.9 (4.5) years) and 121 postmenopausal women (median age 51.7 (3.7) years), matched for median BMI (25.8 (6.7) vs. 25.6 (7.8) kg/m^2^). Associations between body fat outcomes and predictors were examined using multivariable linear regression. **Results:** No significant differences were observed between study groups in body composition parameters, including android fat percentage (%), gynoid fat%, total body fat%, or android/gynoid ratio. Unlike age, menopausal status, or years since menopause, BMI was the only significant predictor of body fat distribution. In the entire cohort, total body fat percentage showed a modest but significant positive correlation with age (*ρ* = 0.200, 95%*CI* [0.043, 0.345], *p* = 0.009), while the menopause onset age was positively correlated with BMI (*ρ* = 0.195, 95%*CI* [0.002, 0.369], *p* = 0.032). Among postmenopausal women within the first two years of menopause, the android/gynoid ratio showed a positive correlation with years of estrogen deprivation (*ρ* = 0.451, 95%*CI* [0.144, 0.707], *p* = 0.007). **Conclusions:** Age was correlated with higher total body fat %; neither age nor menopausal status was correlated with BMI. In early postmenopause, the android/gynoid ratio increased with years since menopause. The median age at menopause observed in our study was 48 years, which is lower than in other Caucasian studies.

## 1. Introduction

Excess body weight, due to its increasing global prevalence and its role as a major risk factor for numerous chronic conditions, has become one of the most pressing public health threats [1]. Numerous studies have linked obesity to poorer quality of life and a wide range of adverse health outcomes, including osteoporosis, fragility fractures, cardiovascular disease (CVD), and increased mortality from all cancers [2,3,4].

Body mass index (BMI), considered the standard for diagnosing obesity, is calculated as the ratio of weight (in kilograms) to the square of height (in meters), but it does not account for the distribution of fat and lean mass [1]. The measurement of waist and hip circumferences, and their corresponding ratio, may improve the assessment of the health impact of body fat and is widely used by healthcare practitioners [5]. However, to assess the relationship between fat distribution and metabolic risk, advanced imaging techniques remain the reference standard in research, despite their limited use in routine clinical practice [5].

Superior to anthropometric measurements, whole-body dual energy X-ray absorptiometry (DXA) is a widely accepted reference method for assessing body composition, due to its low radiation dose and automated analysis algorithms, but it is costlier, more tedious, and has limited accessibility [5,6]. Besides total body fat, DXA can also approximate regional fat distribution, including percentages of android fat—the area around the waist between the mid-lumbar spine and the top of the pelvis, and gynoid fat—the gluteo-femoral area between the femoral heads and mid-thigh [7]. Modern GE Lunar fan-beam systems (such as the Prodigy and iDXA) paired with enCORE analysis software provide measurements of total body and regional fat, with excellent in vivo precision and high reproducibility between scans [8]. Leg fat mass and gynoid fat distribution are presumed to have a protective association with CVD risk markers and reduced odds of elevated blood glucose [9], whereas android fat distribution is linked with insulin resistance [10]. According to some studies, the android/gynoid ratio features a stronger correlation with cardiometabolic risk than the gynoid fat percentage, android fat percentage, or total body fat [9]. Regional iDXA hip and lumbar scans, which are broadly used for evaluating bone mineral density and bone mineral content, especially in postmenopausal women, also provide percentages of regional android and gynoid fat distribution through the enCORE software [11]. This information may be advantageous when a more extensive scan is not feasible, as the resulting fat distribution parameters have been previously validated against whole-body DXA in females using the same DXA system and software, showing moderate-to-high accuracy in estimating whole-body android, gynoid, or total body fat [11]. Although the official software’s predicting regional body fat algorithms are closed-source, Lestie et al. developed and validated prediction equations from paired lumbar-spine, hip, and whole-body scans in a large clinical sample and reported good agreement between predicted and measured body fat parameters [12].

Excess body weight is more prevalent in women than in men, globally and nationally [13,14]. A recent study of a cohort of 2103 Romanian adults has consistently shown a higher prevalence of obesity among women, particularly in middle and older age [13]. The prevalent accumulation of adipose tissue in women during midlife has been ascribed to several mechanisms, including aging, a diminished metabolic rate due to sarcopenia, lower physical activity, and hormonal changes linked to the menopausal transition [3].

Abdominal fat, an endocrine organ that produces many adipokines and substances associated with insulin resistance, metabolic syndrome, and type 2 diabetes mellitus, can be somewhat quantified by the visceral distribution of adipocytes. These adipocytes also promote inflammation, a key risk factor for cardiovascular and metabolic diseases [4]. Consequently, overweight women with central obesity have a similar mortality risk to normal BMI women who also have central fat accumulation, in terms of hypertension, dyslipidemia, and cardiovascular mortality [15].

The 2012 Executive Summary of the Stages of Reproductive Aging Workshop (STRAW+10) defined menopause as the final menstrual period and restructured the early postmenopausal period, distinguishing the first two years after menopause onset as stages +1a and +1b due to their importance in stabilizing new hormonal levels [16]. Some studies suggest that the continuous decline in endogenous estrogen levels during the perimenopausal period affects body fat mass and distribution, leading to increased central fat in postmenopausal women [3]. However, the observed changes in fat mass between pre- and postmenopausal women were attributed primarily to aging, with little additional influence from menopause itself [10]. This has led to controversy over whether menopause or chronological aging, which occurs concurrently with ovarian aging, has a greater effect on fat distribution, rather than on overall body weight or BMI [3,10,17,18,19].

Distinguishing the effects of chronological aging from those of ovarian hormonal decline is challenging, as both processes occur simultaneously, leading to ongoing debate in the literature [3,4,10]. Previous studies have often included wide age ranges, lacked BMI-matched pre- and postmenopausal groups, and provided limited data from Eastern European populations. Consequently, there is a need for focused investigations using common but precise imaging techniques, such as regional DXA scans, in narrowly defined age groups, to clarify how aging and menopausal transition independently and jointly influence body fat parameters. Using radiologic imaging techniques, our cross-sectional study aimed to clarify these relationships. We hypothesized that aging is associated with increases in overall body fat mass, whereas menopausal status is linked to more specific changes in regional fat distribution, such as android fat predominance. The main aim of this study was to compare total body fat and regional fat distribution in BMI-matched pre- and postmenopausal women using hip and lumbar DXA scans. A secondary objective was to determine the possible associations between body fat parameters and age and BMI across different menopausal stages as defined by STRAW+10. Given the paucity of data in Romanian populations, another objective was to determine the age at menopause onset in our cohort and to examine its relationship with BMI and fat mass.

## 2. Materials and Methods

### 2.1. Study Design and Participants

This observational, cross-sectional study included community-based, clinically healthy women aged 45–55 years who presented consecutively to Elias Emergency University Hospital, Bucharest, Romania, between December 2018 and December 2019. All women meeting these criteria were initially screened, and this consecutive recruitment strategy maximized the pool of potential participants and ensured that comparisons primarily reflected differences in menopausal status rather than chronological age or BMI. The 45–55-year age range was selected to minimize the confounding effect of chronological aging while focusing on the perimenopausal and early postmenopausal period, centered around the mean age of menopause (~50 years). In total, 289 women within this target age range who underwent lumbar spine and hip DXA scanning during the study period were initially screened. Baseline demographic and anthropometric data were collected for all screened individuals, including date of birth, scan date, height, weight, and age at menopause onset. Relevant medical history was also reviewed to confirm eligibility. Menopause onset age was determined anamnestically, based on the absence of menstrual periods for 12 consecutive months. All participants provided written informed consent for the use of their medical records and for the publication of the study.

Women were excluded if they met any of the following criteria: menopause onset before the age of 45 (N = 16), more than 10 years since menopause (N = 22), prior glucocorticoid exposure (N = 5), surgical menopause (N = 32), or use of hormonal therapy (N = 11). These criteria were applied to minimize potential confounding and led to the exclusion of 86 participants.

**Figure 1:**
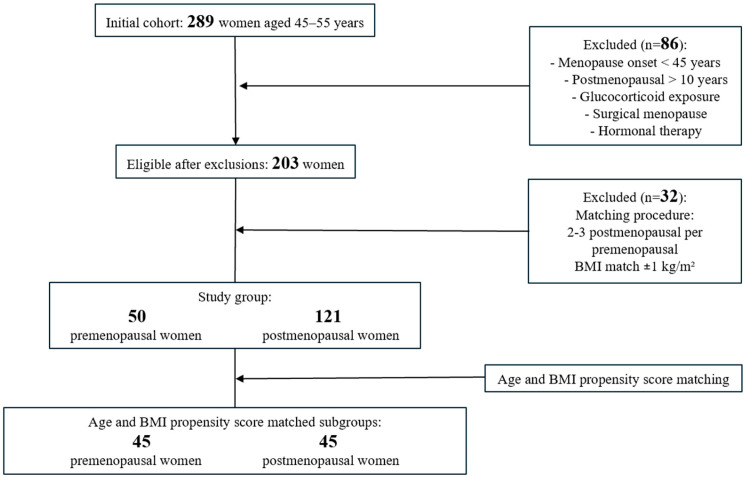
Flow diagram of the study participants.

Within the remaining eligible participants, the next step was a neighbor-matching technique applied for BMI (±1 kg/m^2^), manually assigning two to three postmenopausal women to each premenopausal participant [20]. This approach was intended to minimize differences in body fat related to obesity and led to the exclusion of 7 pre- and 25 postmenopausal women who presented with outlier BMI values. The final analytic cohort consisted of 50 premenopausal and 121 postmenopausal women (Figure 1). Further age and BMI propensity score matching was applied to generate two strictly matched subgroups (45 pre- and 45 postmenopausal women) for comparative analysis.

### 2.2. Body Composition Assessment

Patients underwent hip and lumbar scans exclusively, performed by the same experienced operator, using the dual-energy X-ray absorptiometer (Prodigy, GE Healthcare Lunar, Madison, WI, USA). This dual-energy X-ray absorptiometer differentiates bone, lean tissue, and fat mass using two photon energies, providing precise regional measurements of the hip and lumbar spine; whole-body DXA composition scans were not available. Based on internally developed, proprietary algorithms that are not publicly disclosed, the enCORE software (version 16, GE Healthcare, Madison, WI, USA) was used to provide estimates of body composition parameters, such as total body fat, android fat, and gynoid fat percentages, derived from lumbar and hip scans [12]. Standard procedures for quality control were followed to ensure accuracy and reproducibility [21].

### 2.3. Ethical Considerations

Being an observational study, its design and reporting followed the Strengthening the Reporting of Observational Studies in Epidemiology (STROBE) guidelines [22]. All procedures complied with the principles of the Helsinki Declaration (2013) and were approved by the Elias Emergency University Hospital Ethics Committee 9299/2 October 2017. Patient confidentiality was protected, and all data were de-identified before analysis.

### 2.4. Statistical Analysis

Data analysis was conducted using SPSS software, version 28 (IBM, Armonk, NY, USA), with statistical significance set at *p* < 0.05. Shapiro–Wilk normality test and graphical methods (histograms, quantile–quantile plots) were used to determine the normality of the distribution of continuous variables. Continuous variables are expressed as mean ± standard deviation (SD) for normally distributed data or as median (IQR, interquartile range) for nonparametric data, both followed by 95% confidence intervals (95%*CI*s) [Lower, Upper]. Group comparisons for parametric variables used the independent-sample *t*-test, whereas nonparametric variables were compared with the Mann–Whitney test. Effect size (Cohen’s *d* for *t*-tests, or *ϕ* for Mann–Whitney tests) and corresponding 95%*CI* were calculated and reported alongside the test statistics (*t* or *Χ*^2^). Categorical variables are expressed as numbers (percentages) and summarized in contingency tables, with test statistics, Cramer’s *V*, and 95%*CI* reported. Associations between variables were assessed using Spearman’s correlation coefficients (rho, *ρ*) and reported with 95%*CI*.

To reduce the influence of BMI on fat distribution, propensity score matching (PSM) was applied to the postmenopausal group using BMI and age as covariates, implemented in SPSS software (version 28, IBM, Armonk, NY, USA). The caliper width was set at 0.023, calculated as 0.2 × SD of the logit (0.13) of the estimated probability derived from a binary logistic regression [23]. This procedure resulted in matched subgroups of 45 pre- and postmenopausal women. Standardized mean differences for age and BMI between the propensity score-matched subgroups are presented in Table 1.

Separate multivariable linear regression models were fitted for total body fat %, android fat %, gynoid fat %, and the android/gynoid ratio. In the full cohort, predictors included age, BMI, and menopausal status (pre/post). In the postmenopausal subgroup, years since menopause replaced menopausal status. All predictors were entered simultaneously. Model assumptions (linearity, normality of residuals, and homoscedasticity) were verified. Regression coefficients (*B*), standardized coefficients (*β*), standard errors (SE), *t*-values, *p*-values, 95% confidence intervals, and partial *R*^2^ are reported. Overall model fit was summarized using adjusted *R*^2^, standard error of the estimate (*SEE*), *F*-statistic, and associated *p*-value.

Initial BMI-group matching ensured comparable BMI distributions for primary analyses. Propensity score matching (PSM) was applied as a sensitivity analysis to also balance age and BMI between pre- and postmenopausal subgroups.

## 3. Results

### 3.1. Participant Characteristics and Comparative Body Fat Distribution

A total of 171 participants were included in our study, comprising 50 healthy, middle-aged, premenopausal women (median age of 47.9 (4.5) years) and 121 postmenopausal women (median age of 51.7 (3.7) years). There was no statistical difference in BMI between groups (premenopausal: 25.8 (6.7) kg/m^2^, postmenopausal: 25.6 (7.8) kg/m^2^). Median values of total body fat percentage, android fat percentage, gynoid fat percentage, and android/gynoid ratio did not differ between pre- and postmenopausal groups (Table 2).

For a more detailed comparison, we analyzed 45 premenopausal women versus 45 age- and BMI-propensity-matched postmenopausal controls. Total body fat percentage, gynoid fat percentage, android fat percentage, and android/gynoid ratio were similar between the propensity score-matched subgroups, as seen in Table 3, with no statistical difference in the distribution of body fat.

### 3.2. BMI Categories

The distribution of BMI categories showed no statistically significant difference between pre- and postmenopausal women: *Χ*^2^(5, 171) = 4.044, *p* = 0.539, with a small effect size, Cramer’s *V* = 0.15, 95%*CI* [0.01, 0.30]. Among premenopausal women, 42% were normal weight (BMI 18.5–24.9 kg/m^2^), compared to 41.3% of postmenopausal women. Overweight or obese women (BMI ≥ 25 kg/m^2^) comprised 56% of premenopausal and 57% of postmenopausal women (Table 4).

### 3.3. Menopause Onset and Duration

**Figure 2:**
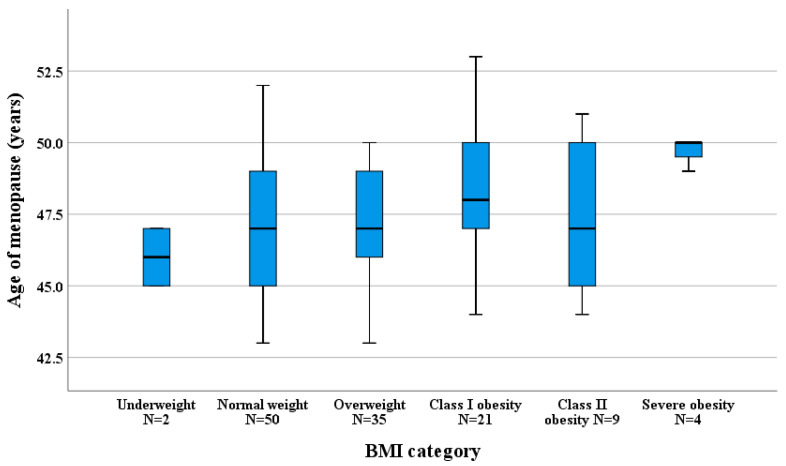
Median age at menopause across BMI categories.

The median age of menopause onset was 48 (4) years, with a median duration of 3 (3) years of estrogen deprivation. When stratified by BMI, the median age of menopause onset was 47 years for normal and overweight women, 48 years for women with class I obesity, 47 years for women with class II obesity, and 50 years for women with a BMI higher than 40 kg/m^2^ (Figure 2). Unlike findings reported in the literature [24], there was no statistical difference in median menopause onset age between normal weight and obese women (47 (4) vs. 48.5 (3) years, *Χ*^2^(1, 84) = 1.538, *ϕ* = 0.16, 95%*CI* [0.00, 2.00]), *p* = 0.215, probably due to the small number of patients in some categories.

### 3.4. Correlations with Age and Body Composition

In the entire study cohort, age was positively correlated with total body fat percentage (*ρ* = 0.200, 95%*CI* [0.043, 0.345], *p* = 0.009), but not with BMI (*ρ* = 0.150, 95%*CI* [0.070, 0.280], *p* = 0.051). This correlation with total body fat percentage remained significant in the BMI propensity-matched subgroups (*ρ* = 0.255, 95%*CI* [0.064, 0.429], *p* = 0.010). Menopause onset age was positively correlated with BMI (*ρ* = 0.195, 95%*CI* [0.002, 0.369], *p* = 0.032), but showed no significant association with total body, android, gynoid fat percentage, nor with the android/gynoid ratio.

### 3.5. STRAW+10 Staging Analysis

When the postmenopausal women were classified according to the STRAW+10 staging system, menopause onset age positively correlated with gynoid fat percentage in stage +1c (*ρ* = 0.230, 95%*CI* [0.009, 0.449], *p* = 0.040). Associations between years since menopause and body composition parameters across STRAW+10 postmenopausal stages are summarized in Table 5. Overall, no significant correlations were observed for BMI, total body fat percentage, or regional fat compartments.

**Figure 3:**
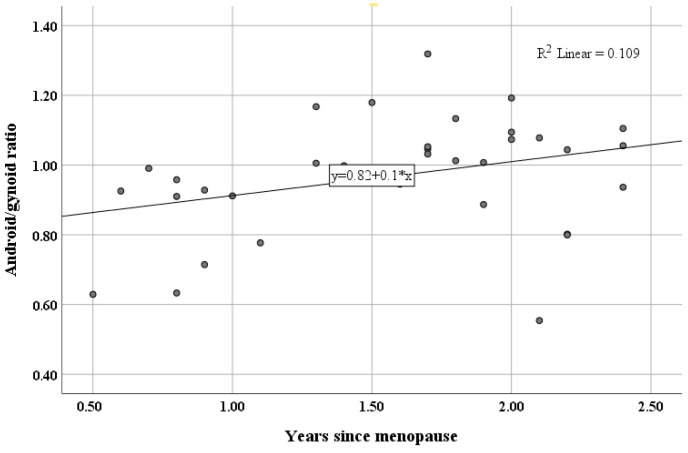
Correlation of the android/gynoid ratio with years since menopause in STRAW+10 stages +1a and +1b.

In contrast, among women in stages +1a and +1b (the first two years postmenopause), the android/gynoid ratio was the only body composition parameter significantly correlated with years since menopause (*ρ* = 0.451, 95%*CI* [0.144, 0.707], *p* = 0.007), as shown in Figure 3.

### 3.6. Effects of Age, BMI, and Menopausal Status on Body Fat Parameters

Multivariable regression in the full cohort showed that BMI was the only significant predictor of total body fat %, android fat %, gynoid fat %, and A/G ratio (Table 6). Neither age nor menopausal status independently predicted these outcomes. In the postmenopausal subgroup, years since menopause also did not significantly predict any body fat outcome, confirming that BMI is the primary determinant of body fat distribution in this sample (Table 7).

Correlation analyses suggested weak associations between age and total body fat %, which were not independent of BMI, highlighting the importance of multivariable regression for separating confounding effects.

## 4. Discussion

The aim of this study was to evaluate differences in DXA-derived total and regional body fat parameters between 50 pre- and 121 postmenopausal women aged 45–55 years while minimizing the confounding effect of BMI. In this BMI-matched cross-sectional analysis, no statistically significant differences were observed between groups in total body fat, android, or gynoid fat percentages. Multivariable analysis indicated that BMI, rather than menopausal status or age, was independently associated with body fat parameters. Weak but statistically significant correlations were identified between age and total body fat, age at menopause onset and BMI, and, among women in early postmenopause, between years since menopause and the android/gynoid ratio.

In our cohort of midlife women, the prevalence of overweight and obese participants was 56.8%, with 55.2% of premenopausal and 57% of postmenopausal women having a BMI greater than 25 kg/m^2^. As noted in multiple reports, women tend to gain weight with age regardless of the menopausal transition; even when lifestyle interventions influence BMI, age independently impacts total body fat percentage [3].

Although menopause is recognized as a challenging period for maintaining weight due to fatigue and vasomotor symptoms (VMSs) [2,3,4,19], our age- and BMI-propensity-matched subgroups showed no statistically significant differences in body fat composition parameters between pre- and postmenopausal women. Unlike menopause, age showed a modest but significant association with increased total body fat percentage (*ρ* = 0.255, 95%*CI* [0.064, 0.429], *p* = 0.011). Contrasting with studies showing menopause as a significant predictor of decreased lean mass and increased body fat percentage [23,24], our multivariable linear regression models indicated that BMI, rather than age, menopausal status, or years since menopause, was the only significant predictor for body fat parameters.

Our findings can be interpreted in the context of previous DXA-based body composition studies. Imboden et al. proposed reference standards for total and regional fat using GE Healthcare Lunar DXA in Caucasian adults and demonstrated that body fat parameters are strongly influenced by overall adiposity with age [7]. In line with these observations, our results showed that BMI was the primary determinant of total and regional fat parameters, while menopausal status was not independently associated with body fat distribution. Unlike this population-based study, which includes wide age ranges [7], our analysis focused on BMI-matched women within a narrow menopausal transition window. This may explain the weaker age and menopause- related associations observed.

In addition, intervention data from Ljubojević et al. indicate that changes in body composition over short periods are modest and closely linked to overall adiposity rather than to hormonal status alone [25]. This supports the interpretation that lifestyle- or weight-related factors may exert a stronger influence on fat parameters than menopausal status per se, particularly in clinically healthy women during the menopausal transition. Taken together, these studies provide a context for our findings, suggesting that the modest menopause-related associations observed in our cohort should be interpreted against the background of normal age- and adiposity-related variability in body fat distribution.

Compared to a SWAN sub-study [19] evaluating body composition and weight changes in 559 white women during the menopause transition, our cohort had a higher mean body fat percentage (42.05% vs. 40.1%) at similar premenopausal ages (47.9 vs. 46.6 years in SWAN) and lower BMI for the Romanian participants (25.6 kg/m2 vs. 27.7 kg/m^2^). These differences may reflect population-specific lifestyle, environmental, and genetic factors.

Previous studies suggested that a higher BMI is correlated with later menopause onset in various ethnicities [26]. We observed a similar positive correlation between BMI and menopause onset age (*ρ* = 0.195, 95%*CI* [0.002, 0.369], *p* = 0.032), which may reflect higher levels, adipokine-mediated effects on ovarian or hypothalamic function, or genetic variants influencing estradiol decline during the menopausal transition [24,27]. Not only weight but also genetic and demographic factors, alongside neuroendocrine aging through multiple gene interactions [28,29], can influence menopause timing [14]. Lifestyle, reproductive history, general health, and even prenatal factors, such as ovarian reserve, may also play a role [30,31,32].

In our study, menopause onset occurred at a median age of 48 years, approximately three years earlier than reported in the literature [33]. This younger age may reflect ethnic characteristics, as another Romanian study reported a similar mean age at menopause in 364 postmenopausal women with osteoporosis [34]. It also underlines the need for larger cohort studies that can differentiate ethnic characteristics from potential confounders like nutrition, reproductive history, physical activity, and other regional health determinants, to accurately evaluate menopausal timing and its metabolic impact within the context of demographic and environmental factors.

Age at menopause onset positively correlated with gynoid fat % during stage +1c of the STRAW+10 system (3rd–8th year of the postmenopause period; *ρ* = 0.230, 95%*CI* [0.009, 0.449], *p* = 0.040), suggesting that longer estrogen exposure may help preserve this fat distribution even in early postmenopause. In contrast, the android/gynoid ratio correlated positively with years of menopause in stages +1a and +1b (first two years postmenopause), indicating a critical window for central fat accumulation. These observations may help explain the increased cardiometabolic risks in postmenopausal women [35]. Considering that androgens in postmenopause increase abdominal adipocyte size and waist/hip ratio [36], these changes likely reflect the impact of perimenopausal hormonal fluctuations on body composition [4].

Our study’s strengths include assessing body fat distribution in BMI-matched pre- and postmenopausal women and analyzing less commonly reported body fat parameters from regional DXA scans, which may provide valuable information on central fat accumulation in early postmenopausal women during bone density assessments. In addition to sex, age, height, weight, and BMI, hip and lumbar DXA scans may predict trunk fat mass with up to 94% accuracy and whole-body fat mass with up to 95% accuracy, both of which are linked to cardiometabolic outcomes [12]. These scans are commonly used for screening bone mineral density, but further research is needed to evaluate their potential to replace the current gold standard for assessing body composition, the whole-body DXA scan, which involves higher costs and increased radiation exposure. Considering the median age of the premenopausal women (47.9 years) and the median age at menopause onset (48 years), this subgroup likely captured the last 1–3 years of perimenopause, or late menopausal transition, according to the STRAW+10 stages. The postmenopausal subgroup had a median age of 51.7 years, with a median of 3 years since menopause, representing early postmenopause. Therefore, our study analyzed a well-balanced cohort across the menopausal transition.

The study has several limitations. First, the age at menopause onset was determined only through anamnestic assessment; estradiol and follicle-stimulating hormone levels were not measured in our cohort. Although reliance on self-report may introduce some misclassification, previous research indicates that self-reported age at menopause is reasonably accurate in population-level settings [37]. Second, the cross-sectional design limits causal inference, as it cannot distinguish age-related changes from menopausal-related effects; nonetheless, it allowed us to examine participants at different menopausal stages simultaneously, providing a broad overview of body composition patterns across the menopausal transition. Third, several potentially relevant confounding variables, such as smoking status, physical activity, diet, and history of depression, were not collected, which may have influenced some associations.

Fourth, data on visceral adipose tissue, lean body mass, and leg fat mass were not available, as our body composition parameters were derived from hip and lumbar DXA scans rather than whole-body scans. A 2007 study in a southern Chinese population using Lunar DXA with enCORE software analyzed the accuracy of regional DXA scans (hip and lumbar) in predicting total body fat percentage, as well as gynoid and android fat and their ratio, in 630 females, aged 20–87 [11]. Prediction equations incorporating age, BMI, and lumbar and hip scans explained up to 84% of the variance in total body fat percentages from whole-body scans and demonstrated high accuracy during cross-validation (*R*^2^ = 0.85). Although lumbar spine percentage correlated strongly with whole-body android fat percentages (*r* = 0.92) and left hip percentages correlated with whole-body gynoid fat percentages (*r* = 0.75), the combined regional DXA prediction equation accounted for only 63% of the variation in the whole-body android/gynoid ratio, indicating satisfactory but limited accuracy [8]. Given that these results were derived from a southern Chinese population, and that the enCORE software equations predicting total body, android, and gynoid fat percentages from regional scans are proprietary and not publicly available, the absence of whole-body scan data may limit the precision of some body composition estimates. Despite these limitations, regional hip and lumbar DXA scans, validated in a comparable population, still provide reasonably reliable estimates of fat distribution, allowing our study to offer meaningful insights into body composition patterns during the menopausal transition.

Interpretation of our findings should consider important methodological differences with existing DXA-based studies. Most references or population-based analyses, including those using GE Lunar systems, encompass wide age ranges and heterogeneous BMI distributions, which tend to amplify age- and menopause-related associations with body fat parameters. In contrast, our study deliberately focused on a narrow age window during the menopausal transition and applied BMI matching to minimize adiposity-related confounding. This design choice likely contributed to the modest magnitude of menopause-related associations observed and may explain discrepancies with studies reporting more pronounced differences. The limited availability of comparable datasets highlights both the constraints and the specificity of our findings.

Additionally, our study was conducted in a single Endocrinology Referral Center, which may have introduced selection bias and limited transferability when comparing our findings to Western populations. The earlier menopause onset observed in our cohort may reflect specific population characteristics or referral-center patterns; confirmation in population-based samples is warranted. Finally, the relatively small number of patients reduced statistical power for comparisons of total body fat, gynoid and android fat percentages, and the android/gynoid ratio between pre- and postmenopausal groups. While we acknowledge that our study may be underpowered to detect very small differences, the consistency of our results across multiple analytical approaches allows us to state that, in this cohort, no identifiable effect of menopause on body fat distribution was detected, beyond that explained by BMI. However, the study still captured stage-specific body composition data, providing useful insights into trends in fat distribution across the menopausal transition.

Future research should follow women from perimenopause through postmenopause to better characterize changes in body composition. Examining different fat compartments alongside lifestyle and hormonal factors may help identify strategies to maintain a healthy weight and fat distribution. Insights into genetic and epigenetic influences could provide valuable information to explain variations in menopause timing and their impact on body composition. Considering population-specific characteristics may help design tailored interventions to reduce cardiometabolic risk during and after the menopausal transition. Additionally, assessing changes in fat distribution during the early postmenopausal years could help identify critical periods for preventive measures.

## 5. Conclusions

In this Romanian study of clinically healthy women aged 45–55 years, no statistically significant differences in regional DXA-derived body fat parameters were observed between BMI-matched pre- and postmenopausal subgroups. Across the cohort, BMI was the only independent predictor of total, android, and gynoid fat parameters, as well as the android/gynoid ratio, whereas age and menopausal status showed no independent association with body fat distribution.

Secondly, age showed a weak but statistically significant positive correlation with total body fat percentage, without a corresponding association with BMI. Among postmenopausal women within the first two years after menopause, the android/gynoid ratio showed a weak positive correlation with years since menopause, consistent with estrogen deprivation. Additionally, age at menopause onset was weakly positively correlated with BMI. The median age of menopause onset in this cohort was 48 years.

Taken together, these findings suggest that, within the limitations of a cross-sectional design and modest sample size, overall adiposity rather than menopausal status appears to be the predominant determinant of body fat distribution during the menopausal transition, while age- and menopause- related associations were small in magnitude. Prospective studies are required to clarify whether small longitudinal changes in fat distribution occur beyond those attributable to BMI.

## Figures and Tables

**Table 1:** Covariate balance between groups before and after propensity score matching for age and BMI.

Variable	Premenopause (Before PSM, N = 50) Mean ± SD	Postmenopause (Before PSM, N = 121) Mean ± SD	SMD (Before PSM)	Premenopause (After PSM, N = 45) Mean ± SD	Postmenopause (After PSM, N = 45) Mean ± SD	SMD (After PSM)
Age (years)	48.8 ± 2.65	51.36 ± 2.81	0.92	49.07 ± 2.64	49.11 ± 2.53	0.01
BMI (kg/m^2^)	26.83 ± 5.32	27.25 ± 5.95	0.07	26.65 ± 5.11	26.07 ± 5.81	0.10

Note: SMD values < 0.1 indicate negligible imbalance; values for age and BMI in each group are presented as means ± SD.

**Table 2:** Participant characteristics and body composition parameters.

Parameter	Premenopause (N = 50)	Postmenopause (N = 121)	Test Statistics, Effect Size, 95%*CI*
Age (years)	47.9 (4.5), 95%*CI* [47.1, 50]	51.7 (3.7), 95%*CI* [50.83, 51.91]	***Χ*^2^ (1,171) = 21.230, *ϕ* = 0.35, 95%*CI* [−4.00, −2.00]**
BMI (kg/m^2^)	25.8 (6.7), 95%*CI* [24.0, 28.7]	25.6 (7.8), 95%*CI* [24.8, 26.6]	*Χ*^2^(1, 171) = 0.002, *ϕ* = 0.01, 95%*CI* [−1.90, 1.40]
Total Body Fat %	42.05 (12.75), 95%*CI* [38.40, 45.40]	44.1 (11.25), 95%*CI* [42.00, 45.60]	*Χ*^2^(1, 171) = 0.389, *ϕ* = 0.04, 95%*CI* [−3.70, 1.50]
Gynoid Fat %	47.15 (5.20), 95%*CI* [45.96, 48.20]	47.05 (6.70), 95%*CI* [46.00, 48.40]	*Χ*^2^(1, 171) = 0, *ϕ* = 0.00, 95%*CI* [−1.70, 1.70]
Android Fat %	45.20 (15.8), 95%*CI* [42.55, 48.85]	47.85 (12.60), 95%*CI* [45.50, 50.25]	*Χ*^2^(1, 171) = 1.020, *ϕ* = 0.07, 95%*CI* [−4.90, 1.80]
Android/Gynoid Ratio	0.93 (0.24), 95%*CI* [0.88, 1.03]	0.99 (0.22), 95%*CI* [0.94, 1.04]	*Χ*^2^(1, 171) = 1.020, *ϕ* = 0.07, 95%*CI* [−0.08, 0.02]

**Bold means statistically significant results.** Values are estimates derived from regional hip and lumbar DXA scans. Median (IQR) values are shown, with 95% confidence intervals. The Mann–Whitney test was used for median comparisons.

**Table 3:** Demographic characteristics of age- and BMI-matched groups using propensity score matching.

Parameter	Premenopause (N = 45)	Postmenopause (N = 45)	Test Statistics, Effect Size, 95%*CI*
Age (years)	50 (4), 95%*CI* [47, 50]	49 (4), 95%*CI* [48, 50]	*Χ*^2^(1, 90) = 0.178, *ϕ* = 0.04, 95%*CI* [−1.00, −1.00]
BMI (kg/m^2^)	26 (6.7), 95%*CI* [24, 27.2]	24.9 (6.6), 95%*CI* [23.8, 26.3]	*Χ*^2^(1, 90) = 1.111, *ϕ* = 0.11, 95%*CI* [−1.20, 3.00]
Total Body Fat %	42.70 (12.00), 95%*CI* [39.28, 46.53]	43.00 (14.45), 95%*CI* [39.45, 45.40]	*Χ*^2^(1, 90) = 0.044, *ϕ* = 0.02, 95%*CI* [−2.70, 3.70]
Gynoid Fat %	47.60 (4.80), 95%*CI* [46.70, 47.90]	46.60 (7.90), 95%*CI* [44.60, 49.40]	*Χ*^2^(1, 90) = 0.400, *ϕ* = 0.06, 95%*CI* [−1.50, 3.10]
Android Fat %	45.20 (16.20), 95%*CI* [42.00, 49.50]	46.40 (18.50), 95%*CI* [42.50, 50.50]	*Χ*^2^(1, 90) = 0.044, *ϕ* = 0.02, 95%*CI* [−4.30, 4.30]
Android/Gynoid Ratio	0.92 ± 0.15, 95%*CI* [0.80, 0.98]	0.94 ± 0.18, 95%*CI* [0.88, 0.99]	*t* (88) = −0.289, Cohen’s *d* = −0.06, 95%*CI* [−0.08, 0.06]

Values are estimates derived from regional hip and lumbar DXA scans. Median (IQR) or mean ± SD is shown, with 95% confidence intervals. The Mann–Whitney test was used for median comparisons, and an independent-sample t-test was used for mean comparisons.

**Table 4:** Distribution of BMI subgroups in pre- and postmenopausal women.

BMI Subgroup	Premenopause (N = 50)	Postmenopause (N = 121)
Underweight	1 (2%)	2 (1.7%)
Normal weight	21 (42%)	50 (41.3%)
Overweight	16 (32%)	35 (28.9%)
Class I obesity	10 (20%)	21 (17.4%)
Class II obesity	0 (0%)	9 (7.4%)
Severe obesity	2 (4%)	4 (3.3%)

**Table 5:** Correlations of years since menopause across STRAW+10 stages.

Parameter	First Two Years of Menopause (Stages +1a, +1b) N = 35	3rd–8th Year of Menopause (Stage +1c) N = 81
BMI (kg/m^2^)	*ρ* = 0.313, *p* = 0.067 95%*CI* [−0.006, 0.610]	*ρ* = −0.134, *p* = 0.235 95%*CI* [−0.367, 0.096]
Total Body Fat %	*ρ* = 0.193, *p* = 0.266 95%*CI* [−0.158, 0.521]	*ρ* = −0.129, *p* = 0.250 95%*CI* [−0.344, 0.091]
Gynoid Fat %	ρ = −0.012, *p* = 0.947 95%*CI* [−0.402, 0.379]	*ρ* = −0.099, *p* = 0.383 95%*CI* [−0.332, 0.117]
Android Fat %	*ρ* = 0.246, *p* = 0.154 95%*CI* [−0.085, 0.555]	*ρ* = −0.074, *p* = 0.515 95%*CI* [−0.295, 0.136]
Android/Gynoid Ratio	***ρ* = 0.451, *p* = 0.007** **95%*CI* [0.144, 0.707]**	*ρ* = −0.037, *p* = 0.746 95%*CI* [−0.255, 0.185]

**Bold means statistically significant results.** Pearman’s correlation was used to assess associations between variables.

**Table 6:** Prediction equations from multivariable linear regression models for each body fat parameter in the whole study cohort.

Outcome	Covariate	*B*	*SE*	*β*	*t*	*p*	95%*CI*	Partial *R*^2^	Overall Model Fit
Total body fat %	Intercept	8.9022	6.8244		1.3055	0.194	−4.570, 22.374		adj *R*^2^ = 0.572 *SEE* = 4.976 *F*(3, 167) = 76.706 *p* < 0.001
	Age	0.133	0.142	0.058	0.943	0.347	−0.146, 0.413	0.005	
	**BMI**	**0.989**	**0.067**	**0.748**	**14.650**	**<0.001**	**0.855, 1.122**	0.562	
	Menopausal status	0.157	0.910	0.008	0.172	0.864	−1.640, 1.953	0.001	
Gynoid fat %	Intercept	27.952	5.872		4.760	<0.001	16.358, 39.546		adj *R*^2^ = 0.338 *SEE* = 4.282 *F*(3, 167) = 28.280 *p* < 0.001
	Age	0.093	0.122	0.048	0.766	0.445	−0.147, 0.334	0.003	
	**BMI**	**0.530**	**0.058**	**0.582**	**9.113**	**<0.001**	**0.415, 0.645**	0.332	
	Menopausal status	−0.517	0.784	−0.043	−0.659	0.511	−2.064, 1.031	−0.002	
Android fat %	Intercept	13.689	9.308		1.471	0.143	−4.689, 32.066		adj *R*^2^ = 0.542 *SEE* = 6.787 *F*(3, 167) = 67.568 *p* < 0.001
	Age	−0.083	0.193	−0.028	−0.430	0.668	−0.464, 0.298	−0.001	
	**BMI**	**1.292**	**0.092**	**0.744**	**0.744**	**<0.001**	**1.110, 1.474**	0.541	
	Menopausal status	0.905	1.243	0.042	0.041	0.728	−1.548, 3.358	0.003	
Android/gynoid ratio	Intercept	0.655	0.181		3.624	<0.001	0.298, 1.011		adj *R*^2^ = 0.368 *SEE* = 0.131 *F*(3, 167) = 32.251 *p* < 0.001
	Age	−0.004	0.004	−0.068	−0.976	0.331	−0.011, 0.004	−0.005	
	**BMI**	**0.017**	**0.002**	**0.612**	**9.726**	**<0.001**	**0.014, 0.021**	0.362	
	Menopausal status	0.028	0.024	0.077	1.145	0.254	−0.020, 0.075	0.007	

**Bold means statistically significant results.** Dependent variables: body fat outcomes estimated from regional hip/lumbar DXA scans. Predictors: age, BMI, menopausal status (pre/post). *B*, regression coefficient; *SE*, standard error; *β*, standardized coefficient of regression; partial *R*^2^, proportion of variance in the dependent variable uniquely explained by each predictor, after controlling for other predictors; adj *R*^2^, coefficient of determination, accounts for the number of predictors and the sample size; *SEE*, standard error of the estimate.

**Table 7:** Prediction equations from multivariable linear regression models for each body fat parameter in the postmenopausal group.

Outcome	Covariate	*B*	*SE*	*β*	*t*	*p*	95%*CI*	Partial *R*^2^	Overall Model Fit
Total body fat %	Intercept	13.170	10.091		1.305	0.194	−6.815, 33.156		adj *R*^2^ = 0.545 *SEE* = 5.1699 *F*(3, 117) = 48.928 *p* < 0.001
	Age	0.069	0.212	0.025	0.327	0.744	−0.351, 0.490	0.001	
	**BMI**	**0.955**	**0.081**	**0.742**	**11.760**	**<0.001**	**0.794, 1.116**	**0.541**	
	Years of menopause	0.019	0.273	0.005	0.070	0.944	−0.522, 0.560	0.001	
Gynoid fat %	Intercept	31.563	8.423		3.747	<0.001	14.880, 48.245		adj *R*^2^ = 0.335 *SEE* = 4.314 *F*(3, 117) = 20.107 *p* < 0.001
	Age	0.021	0.177	0.012	0.121	0.904	−0.330, 0.373	0.001	
	**BMI**	**0.513**	**0.068**	**0.583**	**7.559**	**<0.001**	**0.379, 0.648**	**0.329**	
	Years of menopause	0.002	0.228	0.001	0.008	0.994	−0.449, 0.453	0.001	
Android fat %	Intercept	17.552	13.825		1.270	0.207	−9.831, 44.934		adj *R*^2^ = 0.506 *SEE* = 7.081 *F*(3, 117) = 41.608 *p* < 0.001
	Age	−0.117	0.291	−0.033	−0.402	0.688	−0.693, 0.459	0.001	
	**BMI**	**1.225**	**0.111**	**0.726**	**10.992**	**<0.001**	**1.004, 1.446**	**0.509**	
	Years of menopause	0.158	0.374	0.034	0.424	0.673	−0.582, 0.899	0.001	
Android/gynoid ratio	Intercept	0.653	0.263		2.480	0.015	0.131, 1.174		adj *R*^2^ = 0.326 *SEE* = 0.134 *F*(3, 117) = 20.169 *p* < 0.001
	Age	−0.003	0.006	−0.047	−0.496	0.631	−0.014, 0.008	0.002	
	**BMI**	**0.016**	**0.002**	**0.593**	**7.690**	**<0.001**	**0.012, 0.021**	**0.337**	
	Years of menopause	0.003	0.007	0.044	0.464	0.644	−0.011, 0.017	0.001	

**Bold means statistically significant results.** Dependent variables: body fat outcomes estimated from regional DXA scans. Predictors: age, BMI, years since menopause. *B*, regression coefficient; *SE*, standard error; *β*, standardized coefficient of regression; partial *R*^2^, proportion of variance in the dependent variable uniquely explained by each predictor, after controlling for other predictors; adj *R*^2^, coefficient of determination that accounts for the number of predictors and the sample size; *SEE*, standard error of the estimate.

## Data Availability

The data presented in this study are available upon request from the corresponding author due to privacy data being included.

## Abbreviations

The following abbreviations are used in this manuscript, in order of appearance:

CVD	Cardiovascular disease
BMI	Body mass index
DXA	Dual-energy X-ray absorptiometry
VAT	Visceral adipose tissue
STRAW+10	Stages of Reproductive Aging Workshop
STROBE	Strengthening the Reporting of Observational Studies in Epidemiology
SD	Standard deviation
IQR	Interquartile range
95%*CI*	95% confidence interval
PSM	Propensity score matching
*SE*	Standard error
*SEE*	Standard error of the estimate
VMSs	Vasomotor symptoms
